# Supplementary material for: Current Intraoperative Imaging Techniques to Improve Surgical Resection of Laryngeal Cancer: A Systematic Review
Source: Cancers (Basel). 2021 Apr 15;13(8):1895. doi: 10.3390/cancers13081895 (PMC8071167; doi:10.3390/cancers13081895)
Supplement: Supplementary file 1 [file cancers-13-01895-s001.zip › cancers-1177646-supplementary/cancers-1177646-supplementary-for xml/File S1; Search strategy.pdf]

Supplementary File S1 - Search Strategy

Intraoperative imaging for laryngeal cancer

**Database searched**	**via**	**Years of coverage**	**References**	**After de-duplication**
Embase	Embase.com	1971 - Present	2166	2146
Medline ALL	Ovid	1946 - Present	907	140
Web of Science Core Collection	Web of Knowledge	1975 - Present	754	156
Cochrane Central Register of Controlled Trials	Wiley	1992 - Present	101	54
Other sources: Google Scholar (ranked on relevance)			200	141
**Total**			**4128**	**2637**

Embase.com

('epiglottis tumor'/exp OR 'larynx tumor'/exp OR 'larynx'/exp OR (((epiglott* OR larynx* OR microlaryng* OR laryng* OR glott* OR postcricoid* OR subglot* OR supraglot* OR vocal-cord*) NEAR/6 (cancer* OR carcinoma* OR tumor* OR tumour* OR neoplas* OR papilloma* OR granuloma* OR lesion* OR malignan* OR premalignan* OR leukoplak* OR dysplas* OR surg* OR operat*))):ab,ti,kw) AND ('narrow band imaging'/de OR 'autofluorescence'/de OR 'Raman spectrometry'/de OR 'coherent anti Stokes Raman spectroscopy'/de OR 'fluorescence'/de OR 'confocal microscopy'/exp OR 'fluorescent dye'/de OR 'white light'/de OR (((narrowband* OR narrow-band*) NEAR/3 (imag*)) OR autofluorescence* OR auto-fluorescence* OR intrinsic-fluorescence* OR native-fluorescence* OR ((fluorescen* OR imag*) NEAR/3 guided) OR ((light* OR optical* OR molecular*) NEAR/3 (imag*)) OR near-infrared OR tracer* OR target* OR dye* OR probe* OR raman* OR confocal* OR ((contact*) NEAR/3 (endomicroscop* OR microscop* OR endoscop*))):ab,ti,kw) AND ('intraoperative period'/de OR 'intraoperative monitoring'/de OR 'surgery'/exp OR 'surgery':lnk OR 'laryngoscopy'/exp OR (intraoperativ* OR intra-operativ* OR intrasurg* OR surg* OR real-time* OR margin* OR microsurg* OR laryngoscop* OR microlaryngoscop*):ab,ti,kw)

Medline

(Laryngeal Neoplasms/ OR Granuloma, Laryngeal/ OR (((epiglott* OR larynx* OR microlaryng* OR laryng* OR glott* OR postcricoid* OR subglot* OR supraglot* OR vocal-cord*) ADJ6 (cancer* OR carcinoma* OR tumor* OR tumour* OR neoplas* OR papilloma* OR granuloma* OR lesion* OR malignan* OR premalignan* OR leukoplak* OR dysplas* OR surg* OR operat*))).ab,ti,kf.) AND (Optical Imaging/ OR Narrow Band Imaging/ OR autofluorescence/ OR Fluorescence/ OR Microscopy, Confocal/ OR Fluorescent Dyes/ OR (((narrowband* OR narrow-band*) ADJ3 (imag*)) OR autofluorescence* OR auto-fluorescence* OR intrinsic-fluorescence* OR native-fluorescence* OR ((fluorescen* OR imag*) ADJ3 guided) OR ((light* OR optical* OR molecular*) ADJ3 (imag*)) OR near-infrared OR tracer* OR target* OR dye* OR probe* OR raman* OR confocal* OR ((contact*) ADJ3 (endomicroscop* OR microscop* OR endoscop*))).ab,ti,kf.) AND (exp Intraoperative Period/ OR Monitoring, Intraoperative/

OR exp Surgical Procedures, Operative/ OR surgery.fs. OR Laryngoscopy/ OR (intraoperativ* OR intra-operativ* OR intrasurg* OR surg* OR real-time* OR margin* OR microsurg* OR laryngoscop* OR microlaryngoscop*).ab,ti,kf.)

Web of Science

TS=(((((epiglott* OR larynx* OR microlaryng* OR laryng* OR glott* OR postcricoid* OR subglot* OR supraglot* OR vocal-cord*) NEAR/5 (cancer* OR carcinoma* OR tumor* OR tumour* OR neoplas* OR papilloma* OR granuloma* OR lesion* OR malignan* OR premalignan* OR leukoplak* OR dysplas* OR surg* OR operat*)))) AND ((((narrowband* OR narrow-band*) NEAR/2 (imag*)) OR autofluorescence* OR auto-fluorescence* OR intrinsic-fluorescence* OR native-fluorescence* OR ((fluorescen* OR imag*) NEAR/2 guided) OR ((light* OR optical* OR molecular*) NEAR/2 (imag*)) OR near-infrared OR tracer* OR target* OR dye* OR probe* OR raman* OR confocal* OR ((contact*) NEAR/2 (endomicroscop* OR microscop* OR endoscop*)))) AND ((intraoperativ* OR intra-operativ* OR intrasurg* OR surg* OR real-time* OR margin* OR microsurg* OR laryngoscop* OR microlaryngoscop*)))

Cochrane Central

((((epiglott* OR larynx* OR microlaryng* OR laryng* OR glott* OR postcricoid* OR subglot* OR supraglot* OR vocal NEXT cord*) NEAR/6 (cancer* OR carcinoma* OR tumor* OR tumour* OR neoplas* OR papilloma* OR granuloma* OR lesion* OR malignan* OR premalignan* OR leukoplak* OR dysplas* OR surg* OR operat*))):ab,ti,kw) AND ((((narrowband* OR narrow NEXT band*) NEAR/3 (imag*)) OR autofluorescence* OR auto NEXT fluorescence* OR intrinsic NEXT fluorescence* OR native NEXT fluorescence* OR ((fluorescen* OR imag*) NEAR/3 guided) OR ((light* OR optical* OR molecular*) NEAR/3 (imag*)) OR "near infrared" OR tracer* OR target* OR dye* OR probe* OR raman* OR confocal* OR ((contact*) NEAR/3 (endomicroscop* OR microscop* OR endoscop*))):ab,ti,kw) AND ((intraoperativ* OR intra NEXT operativ* OR intrasurg* OR surg* OR real NEXT time* OR margin* OR microsurg* OR laryngoscop* OR microlaryngoscop*):ab,ti,kw)

Google Scholar

"epiglottis|larynx|laryngeal cancer|carcinoma|tumor|tumour|neoplasm" "narrow band imaging"|"optical|molecular imaging"|autofluorescence|"auto|intrinsic|native fluorescence"|tracer|target|dye|probe|raman|confocal intraoperative

Extra search string: Embase.com + head and neck tumor

('epiglottis tumor'/exp OR 'larynx tumor'/exp ***OR 'head and neck tumor'/de*** OR (((epiglott* OR larynx* OR microlaryng* OR laryng* OR glott* OR postcricoid* OR subglot* OR supraglot* OR vocal-cord*) NEAR/6 (cancer* OR carcinoma* OR tumor* OR tumour* OR neoplas* OR papilloma* OR granuloma* OR lesion* OR malignan* OR premalignan* OR leukoplak* OR dysplas* OR surg* OR operat*))):ab,ti,kw) AND ('narrow band imaging'/de OR 'autofluorescence'/de OR 'Raman spectrometry'/de OR 'coherent anti Stokes Raman spectroscopy'/de OR 'fluorescence'/de OR 'confocal microscopy'/exp OR 'fluorescent dye'/de OR (((narrowband* OR narrow-band*) NEAR/3 (imag*)) OR autofluorescence* OR auto-

fluorescence* OR intrinsic-fluorescence* OR native-fluorescence* OR ((fluorescen* OR imag*) NEAR/3 guided) OR ((light* OR optical* OR molecular*) NEAR/3 (imag*)) OR near-infrared OR tracer* OR target* OR dye* OR probe* OR raman* OR confocal* OR ((contact*) NEAR/3 (endomicroscop* OR microscop* OR endoscop*))):ab,ti,kw) AND ('intraoperative period'/de OR 'intraoperative monitoring'/de OR 'surgery'/exp OR 'surgery':lnk OR (intraoperativ* OR intra-operativ* OR intrasurg* OR surg* OR real-time* OR margin* OR microsurg* OR laryngoscop* OR microlaryngoscop*):ab,ti,kw)
