# Supplementary material for: Current Intraoperative Imaging Techniques to Improve Surgical Resection of Laryngeal Cancer: A Systematic Review
Source: Cancers (Basel). 2021 Apr 15;13(8):1895. doi: 10.3390/cancers13081895 (PMC8071167; doi:10.3390/cancers13081895)
Supplement: Supplementary file 1 [file cancers-13-01895-s001.zip › cancers-1177646-supplementary/cancers-1177646-supplementary-for xml/File S2; PRISMA 2020 flow diagram.pdf]

Supplementary File S2 - PRISMA 2020 flow diagram for new systematic reviews

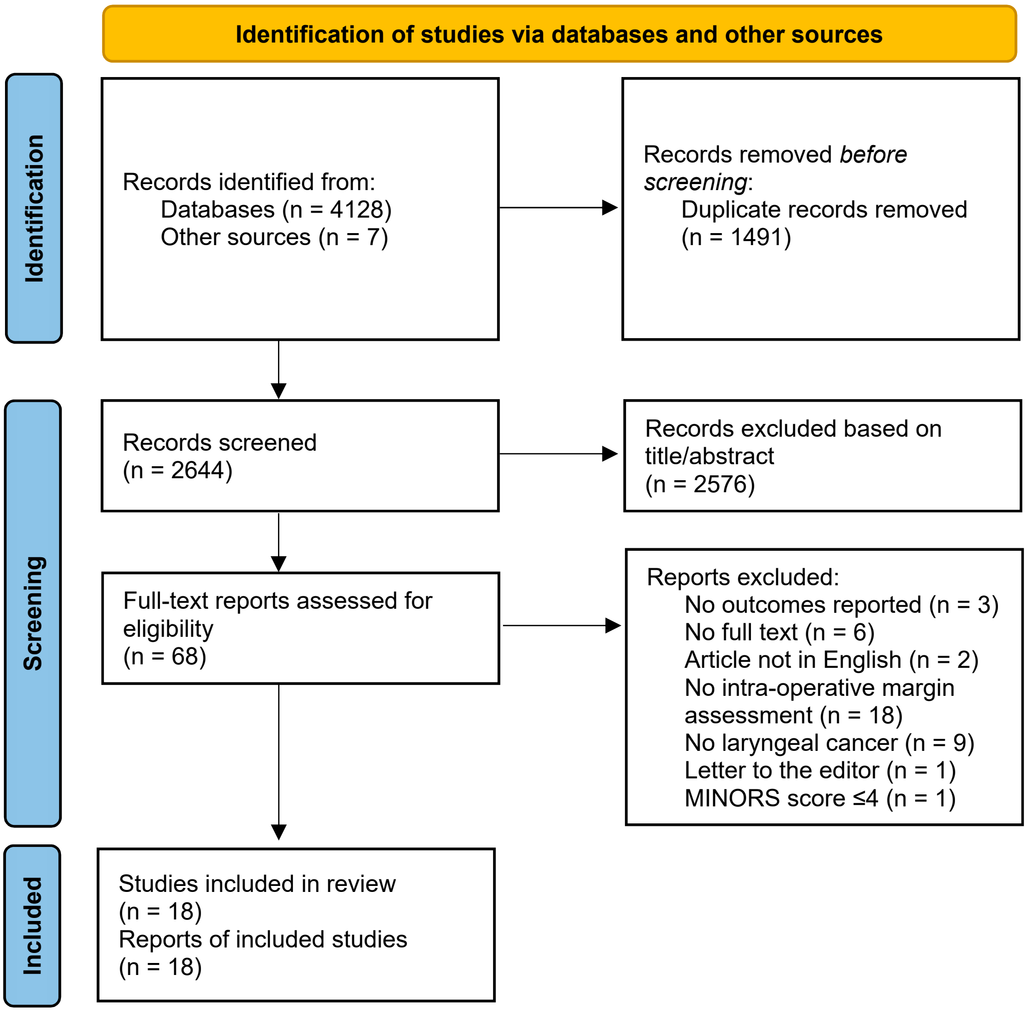

From: Page MJ, McKenzie JE, Bossuyt PM, Boutron I, Hoffmann TC, Mulrow CD, et al. The PRISMA 2020 statement: an updated guideline for reporting systematic reviews. BMJ 2021;372:n71. doi: 10.1136/bmj.n71

For more information, visit: http://www.prisma-statement.org/
