# Supplementary material for: Current Intraoperative Imaging Techniques to Improve Surgical Resection of Laryngeal Cancer: A Systematic Review
Source: Cancers (Basel). 2021 Apr 15;13(8):1895. doi: 10.3390/cancers13081895 (PMC8071167; doi:10.3390/cancers13081895)
Supplement: Supplementary file 1 [file cancers-13-01895-s001.zip › cancers-1177646-supplementary/cancers-1177646-supplementary-for xml/Table S1; MINORS score table.pdf]

Supplementary Table S3 - MINORS Score

Study	Cumulative MINORS score	Maximum MINORS score	Clearly stated aim	Consecutive patients	Prospective data collection	Appropriate endpoint	Unbiased evaluation of endpoints	Appropriate follow-up	Loss to follow-up	Prospective calculation of sample size	Gold Standard control	Contemporary groups	Baseline equivalence of groups	Statistical analysis adapted to study design
Fiz[3]	17	24	2	2	1	2	0	2	1	0	2	1	2	2
Garofolo[18]	18	24	2	2	1	2	0	2	2	0	2	1	2	2
Hainarosie[19]	9	24	0	0	0	1	0	2	2	0	2	1	1	0
Klimza[20]	11	16	2	2	1	2	0	2	2	0	na	na	na	na
Piersiala[21]	12	16	2	2	2	2	0	2	2	0	na	na	na	na
Plaat[22]	17	24	2	2	1	1	0	2	2	0	2	1	2	2
Šifrer[23]	18	24	2	2	2	0	2	2	2	0	2	1	2	1
Srivastava[24]	7	16	0	2	1	0	0	2	2	0	na	na	na	na
Vicini[25]	17	24	2	1	1	2	0	2	2	0	2	2	2	1
Zwakenberg[26]	20	24	2	0	2	2	2	2	2	0	2	2	2	2
Fielding[27]	10	16	2	1	2	1	0	2	2	0	na	na	na	na
Paczona[28]	10	24	2	0	2	0	0	0	2	0	2	1	1	0
Succo[29]	15	24	2	1	2	2	0	2	2	0	2	1	1	0
Csanády[30]	6	16	1	0	0	1	0	2	2	0	na	na	na	na
Digonnet[31]	10	16	1	2	2	1	0	2	2	0	na	na	na	na
Dedivitis[32]	9	16	2	0	2	1	0	2	2	0	na	na	na	na
Stefanescu[33]	7	16	2	0	0	1	0	2	2	0	na	na	na	na
Shakhov[34]	5	16	1	0	0	0	0	2	2	0	na	na	na	na
